# Reliability of popliteal artery flow‐mediated dilation in the seated position

**DOI:** 10.14814/phy2.70252

**Published:** 2025-03-20

**Authors:** Taskina Akhter, Patrick B. Wilson, J. David Branch, Leryn J. Reynolds

**Affiliations:** ^1^ Old Dominion University Norfolk Virginia USA

**Keywords:** endothelial function, prone, reproducibility, vascular function

## Abstract

Flow‐mediated dilation (FMD), a noninvasive measure of endothelial function, is commonly measured in the popliteal artery in the supine and upright body positions. However, reliability studies of the measurement in these positions are not well studied. This study aimed to examine the trial‐retrial and visit‐to‐visit reliability of popliteal artery FMD in the seated position. Popliteal artery FMD was measured in 20 healthy adults across two visits in the seated and prone positions to assess visit‐to‐visit reliability. Two measurements were taken for each body position at each visit to assess trial‐retrial reliability. %FMD was calculated as the percent change from baseline diameter to peak diameter. The reliability of FMD was assessed via the intraclass correlation coefficient (ICC). Popliteal artery %FMD shows moderate‐to‐good reliability in the seated position (ICC: 0.67–0.89) and poor‐to‐moderate reliability in the prone position (ICC: 0.25–0.74) within and between visits. There were no significant differences in baseline diameter or minimum diameter between body positions, visits, or trials (*p* > 0.05). %FMD and peak diameter following cuff deflation demonstrated no significant difference between body positions (*p* > 0.05). Popliteal artery FMD demonstrates good reliability in the seated position, supporting the development of a standardized measurement protocol.

## 1 INTRODUCTION

According to the World Health Organization (WHO), cardiovascular diseases (CVDs) are the leading cause of death globally, claiming an estimated 17.9 million lives each year, which is about 32% of total global deaths (World Health Organization, 2021). A hallmark sign of CVD is impaired endothelial function (Davignon & Ganz, 2004). The endothelium is critical for regulating vascular tone in response to various stimuli (e.g., shear stress) and maintaining vascular health (Green et al., 2004). One noninvasive method of measuring endothelial function is flow‐mediated dilation (FMD), which is used to determine endothelial‐dependent vasodilatory function (Thijssen et al., 2019). This test measures the vasodilatory response of conduit arteries following post‐occlusive reactive hyperemia of a distal limb using high‐resolution Doppler ultrasonography (Green et al., 2014).

It is important for FMD to have high test–retest reliability within and between visits because the application of FMD in conduit arteries, such as the brachial, radial, superficial femoral, and popliteal arteries, has been increasing both in clinical and physiological studies (Betik et al., 2004; Green et al., 2010; Kooijman et al., 2008). Popliteal artery %FMD is utilized both in clinical settings and physiological research for CVD risk and endothelial function evaluation. Several studies on popliteal artery %FMD have been done where the body position of participants varied during the FMD test to include the seated (Liu et al., 2021; O'Brien et al., 2020; Wu, 2022), prone (Kadoguchi et al., 2020; Nakamura et al., 2019; Padilla et al., 2009; Parker et al., 2006), and supine (Morishima et al., 2016, 2017; Rakobowchuk et al., 2011; Restaino et al., 2016; Teixeira et al., 2017) or semi‐recumbent positions (Peddie et al., 2021; Restaino et al., 2015; Vranish et al., 2017). However, the difference in methodological approach in performing popliteal artery %FMD in different studies may impact the physiological responses. For instance, given that shear stress is lower in the upright position compared to the supine position (Schlager et al., 2011) and the important role of shear stress on vascular function (Rubanyi et al., 1986), %FMD may be altered in the seated position compared to the prone position.

The inconsistent use of body position while measuring popliteal artery %FMD in different studies is an important methodological approach which may impact the reliability and comparison of results of different studies. As several studies have measured popliteal artery FMD measurements in the seated position (Liu et al., 2021; O'Brien et al., 2020; Wu, 2022), it is imperative that this methodological approach is reliable. The purpose of this study was to determine the within‐ and between‐visits reliability of popliteal artery %FMD in the seated position. This will provide greater insight into methodological approaches regarding body position when measuring popliteal artery %FMD. It is hypothesized that there would be good reliability of popliteal artery %FMD in the seated position.

## 2 METHODS

### 2.1 Ethics Statement

This study was approved by the Old Dominion University Institutional Review Board. Written informed consent was obtained from all subjects. All experimental procedures and measurements conformed to the Declaration of Helsinki.

### 2.2 Participants

Inclusionary criteria for the study were as follows: 18–45 years of age and nonobese (body mass index (BMI) < 30 kg/m^2^). Further, females were only studied in their early follicular phase to control for the hormonal effects of the menstrual cycle on endothelial function (Williams et al., 2001). Exclusionary criteria were individuals with known CVD, hypertension, or metabolic diseases; taking medication that would impact hemodynamic responses at the time of screening; and females who were pregnant, breastfeeding, or taking any hormonal contraception pills. Eligibility was determined by a health history questionnaire.

### 2.3 Experimental design

This was an experimental, randomized study design and was conducted at the Cardiometabolic Laboratory at Old Dominion University. Following enrollment in the study, participants filled out a health history questionnaire, and height and weight were measured (Detecto, Webb City, MO, USA). The participants then had FMD measured in the seated and prone positions twice within each study visit. Two to 14 days later, participants returned to the laboratory, and the measurements were repeated. A single sonographer performed all FMD measurements and analysis. Further, body composition was assessed via air displacement plethysmography (Bod Pod 2007A, Cosmed USA Inc., Concord, CA).

Flow‐mediated dilation of the popliteal artery was assessed according to the guidelines for the current standardized methodology (Corretti et al., 2002; Thijssen et al., 2019). To minimize confounding factors and their influence on FMD, participants were requested to refrain from engaging in any exercise 24 h prior to the experiments, avoid the consumption of alcohol and caffeine for 12 h, and avoid smoking for at least 6 h beforehand. Experiments were performed in a quiet, temperature‐monitored (21–23°C) room after resting in the seated or prone position for 15 min. Furthermore, as diurnal variation can affect FMD, the repeated measurements were performed at the same time of day (within a 1‐h range) based on recommended guidelines (Thijssen et al., 2019). To minimize the influence of endogenous sex hormones on vascular function, females were evaluated in the early‐follicular phase (i.e., days 1–7 following the onset of menstruation) of their self‐reported menstrual cycle (Thijssen et al., 2011).

Body position (seated or prone) order was randomized using the online randomization tool (www.randomizer.org) for the first trial on each visit to minimize the effect of order. For the subsequent trials in each visit, alternating body positions were assigned. For example, if the first trial was in the prone position based on randomization, the 2nd trial was performed in the seated position, 3rd trial in the prone position, and finally, the 4th trial in the seated position. To measure popliteal artery FMD, the ultrasonography probe was placed immediately distal to the popliteal fossa, and a pneumatic pressure cuff attached to a rapid cuff inflation system (E20 and AG101; Hokanson, Bellevue, WA, USA) was placed around the calf distal to the popliteal fossa (~10 cm) of the right lower limb (Shivgulam et al., 2022). In the seated position, the knee remained at an anatomical 60–70° angle for all measurements, which was assessed using a goniometer. In the prone position, the knee remained at a 0° angle. Baseline arterial lumen diameter and blood velocity were measured for 3 min. While continuing to record arterial lumen diameter and blood velocity, the pressure in the cuff was rapidly inflated to 220 mmHg and remained at that occlusion pressure for 5 min. Then, following deflation of the cuff, another 5 min of continuous ultrasound recording was done. Vascular measurements were recorded via duplex ultrasonography using an 11‐MHz multifrequency linear array probe connected to a high‐resolution ultrasound system (Logiq P9, GE Medical Systems, Milwaukee, WI), which is reliable and valid (Ratcliffe et al., 2017). Popliteal artery velocity was recorded at a pulsed frequency of 4.2 MHz and was corrected using an insonation angle of ~60° with the cursor set to mid‐vessel. Ultrasound images and video were recorded digitally for off‐line analysis using the Elgato video capture software (Munich, Germany). Off‐line analysis of vessel diameter and velocity was performed using Cardiovascular Suite FMD Studio software version 2.8.1 (Quipu, Pisa, Italy). Participants then rested in the seated or prone position for 30 min to allow blood flow and arterial diameter to return to normal resting conditions, and the same procedure as described above was repeated three additional times (Harris et al., 2007), alternating body positions as described above. Also, immediately before and after each FMD measurement, blood pressure (mmHg), and heart rate (HR) (beats per minute) were measured by an automated sphygmomanometer (Welch Allyn ProBP 2000, Skaneateles Falls, New York, USA) with an appropriately sized cuff. Average systolic blood pressure (SBP), diastolic blood pressure (DBP), and HR are reported as an average of the pre and post FMD measurements.

In the 2nd visit, all measurements were repeated utilizing the same pre‐testing and procedural requirements as the first study visit. Following O'Brien et al. (2020), %FMD was quantified using the following equation: [(post‐cuff deflation peak diameter – baseline diameter) ÷ (baseline diameter) × 100]. The baseline diameter (mm) was calculated using 3‐s data smoothing average (Boyle et al., 2013). The peak (mm) and minimum (mm) diameters were determined as the maximal and minimal dilation during 5 min of cuff deflation and 5 min of cuff inflation using 3‐s data smoothing average, respectively.

### 2.4 Statistical analysis

All statistical analyses were performed using IBM SPSS Statistics 28 (IBM, Armonk, NY, USA). Descriptive statistics were reported as the mean ± S.D. (Standard Deviation). The reliability of %FMD, baseline diameter, post‐cuff deflation peak diameter, and the minimum diameter during cuff inflation between trials and visits were assessed using the intraclass correlation coefficient (ICC) (Hendricks & Robey, 1936). These values were calculated using IBM SPSS Statistics 28 based on absolute agreement and a 2‐way mixed effects model. While interpreting ICC values, the following ranges were used where an ICC < 0.5 indicates poor reliability, an ICC value between 0.5 and 0.75 indicates moderate reliability, values between 0.75 and 0.9 indicate good reliability, and an ICC > 0.90 indicates excellent reliability (Koo & Li, 2016). Given the importance of shear stress on endothelial function (Pyke & Tschakovsky, 2005), and that shear stress is different in the supine versus seated position (Trinity et al., 2014), an ANOVA (analysis of variance) was used to determine differences in body position on measures made during the FMD test. A 3‐way ANOVA (body position × visits × trials) was used to determine differences in baseline diameter, minimum diameter during cuff inflation, and systolic and diastolic blood pressure. These values were found to be normally distributed using the Shapiro–Wilk test for normality (*p* > 0.05). However, heart rate, %FMD, and peak diameter were not found to be normally distributed (Shapiro–Wilk test *p* < 0.05). Thus, we ran a Wilcoxon Signed‐Rank Test on body position for these variables to determine statistical significance (*p* < 0.05). Further, we report the mean ± standard deviation, *Z* score, and median values (interquartile range [IQR]) for heart rate, %FMD, and peak diameter. IQR for those measures was determined as the 25th and 75th percentile using the weighted average.

## 3 RESULTS

An overview of characteristics of the 20 individuals (15 males and five females) who participated in the study are provided in Table 1.

**TABLE 1:** Subject characteristics (*n* = 20).

Variables	Data
Age (years)	29 ± 5
Body mass (kg)	71.9 ± 10.1
Height (cm)	168.5 ± 9.1
BMI (kg/m^2^)	25.4 ± 3.1
Body fat (%)	29.4 ± 10.1
Fat‐free mass (%)	70.6 ± 10.1

*Note*: Values are expressed as mean ± S.D. (standard deviation).

Abbreviation: BMI, body mass index.

SBP and DBP for each FMD trial in both prone and seated positions during each visit are presented in Table 2. There were no significant main effects or interactions for body position, visit, or trial on SBP or DBP using a 3‐way ANOVA (*p* > 0.05). Albeit small, there was a significant difference (Wilcoxon Signed Rank Test) for heart rate to be higher in the seated position (65.59 ± 8.82) compared to the prone position (64.18 ± 9.79) (*Z* = −2.28, *p* = 0.023) (prone: median 66.50 (IQR [56.50–71.50]) and seated: median 66.25 (IQR [59.38–72.50])).

**TABLE 2:** Blood pressure across trials and visits.

Visit no.	Body position	Trial no.	Systolic blood pressure (mmHg)	*p* Value	Diastolic blood pressure (mmHg)	*p* Value
1	Prone	1	111 ± 7.2	**Main effects:** Body position: 0.927 Visit: 0.351 Trial: 0.228 **Interactions:** Body position × visit: 0.800 Body Position × trial: 0.761 Visit × trial: 0.847 Body position × visit × trial: 0.968	71 ± 7.2	**Main effects:** Body position: 0.053 Visit: 0.309 Trial: 0.418 **Interactions:** Body position × visit: 0.454 Body position × trial: 0.255 Visit × trial: 0.710 Body position × visit × trial: 0.956
		2	112 ± 7.2		73 ± 7.3	
	Seated	1	111 ± 5.5		75 ± 6.2	
		2	113 ± 7.2		75 ± 7.4	
2	Prone	1	111 ± 7.0		71 ± 5.9	
		2	111 ± 10.6		73 ± 6.0	
	Seated	1	110 ± 8.8		73 ± 6.4	
		2	111 ± 7.8		73 ± 4.9	

*Note*: Values are expressed as mean ± S.D. (Standard Deviation).

The ICC estimates and their 95% confidence intervals (CI) for baseline diameter, minimum diameter during cuff occlusion, peak diameter following cuff deflation, and %FMD in the prone and seated positions are shown in Tables 3 and 4, respectively. Baseline diameter, minimum diameter during cuff inflation, and peak diameter following cuff deflation within and between visits were found to have high ICC estimates (>0.90), indicative of excellent reliability in both seated and prone positions. In the prone position (Table 3), the ICC estimates of %FMD within and between visits demonstrated poor‐to‐moderate reliability (0.25–0.74), while %FMD within visit ICC estimates demonstrated good reliability in the seated position (Table 4) (0.86–0.89). Moreover, in the seated position (Table 4), %FMD between visit ICC estimates demonstrated moderate‐to‐good reliability (0.67–0.87).

**TABLE 3:** Within and between visits ICC values of each FMD trial in the popliteal artery in the prone position.

Within visit measures		Between visit measures		
Variable	ICC (95% CI)	Variable	ICC (95% CI)	
			Visit 2	Visit 2
			Trial 1	Trial 2
%FMD (Visit 1)	0.60 (0.00–0.84)	%FMD (Visit 1 Trial 1)	0.68 (0.23–0.87)	0.35 (−0.53–0.73)
%FMD (Visit 2)	0.25 (−0.99–0.71)	%FMD (Visit 1 Trial 2)	0.48 (−0.36–0.80)	0.74 (0.33–0.90)
Baseline diameter (mm) (Visit 1)	0.99 (0.98–1.00)	Baseline diameter (mm) (Visit 1 Trial 1)	0.99 (0.97–1.00)	0.99 (0.98–1.00)
Baseline diameter (mm) (Visit 2)	1.00 (0.99–1.00)	Baseline diameter (mm) (Visit 1 Trial 2)	1.00 (0.99–1.00)	0.99 (0.98–1.00)
Peak diameter (mm) (Visit 1)	0.99 (0.98–1.00)	Peak diameter (mm) (Visit 1 Trial 1)	0.99 (0.97–1.00)	0.99 (0.95–1.00)
Peak diameter (mm) (Visit 2)	0.99 (0.98–1.00)	Peak diameter (mm) (Visit 1 Trial 2)	0.99 (0.99–1.00)	0.99 (0.98–1.00)
Minimum diameter (mm) (Visit 1)	0.99 (0.97–1.00)	Minimum diameter (mm) (Visit 1 Trial 1)	0.99 (0.97–1.00)	0.99 (0.98–1.00)
Minimum diameter (mm) (Visit 2)	0.99 (0.97–1.00)	Minimum diameter (mm) (Visit 1 Trial 2)	0.98 (0.96–0.99)	0.98 (0.96–0.99)

**TABLE 4:** Within and between visits ICC values of each FMD trial in the popliteal artery in the seated position.

Within visit measures		Between visit measures		
Variable	ICC (95% CI)	Variable	ICC (95% CI)	
			Visit 2	Visit 2
			Trial 1	Trial 2
%FMD (Visit 1)	0.86 (0.64–0.94)	%FMD (Visit 1 Trial 1)	0.87 (0.66–0.95)	0.83 (0.39–0.94)
%FMD (Visit 2)	0.89 (0.72–0.96)	%FMD (Visit 1 Trial 2)	0.81 (0.47–0.93)	0.67 (0.09–0.87)
Baseline diameter (mm) (Visit 1)	0.98 (0.96–0.99)	Baseline diameter (mm) (Visit 1 Trial 1)	0.98 (0.96–0.99)	0.98 (0.96–0.99)
Baseline diameter (mm) (Visit 2)	1.00 (0.99–1.00)	Baseline diameter (mm) (Visit 1 Trial 2)	0.99 (0.97–0.99)	0.98 (0.96–0.99)
Peak diameter (mm) (Visit 1)	0.98 (0.96–0.99)	Peak diameter (mm) (Visit 1 Trial 1)	0.99 (0.97–1.00)	0.98 (0.96–0.99)
Peak diameter (mm) (Visit 2)	1.00 (0.99–1.00)	Peak diameter (mm) (Visit 1 Trial 2)	0.99 (0.99–1.00)	0.99 (0.97–1.00)
Minimum diameter (mm) (Visit 1)	0.97 (0.93–0.99)	Minimum diameter (mm) (Visit 1 Trial 1)	0.98 (0.95–0.99)	0.98 (0.95–0.99)
Minimum diameter (mm) (Visit 2)	1.00 (0.99–1.00)	Minimum diameter (mm) (Visit 1 Trial 2)	0.99 (0.98–1.00)	0.99 (0.98–1.00)

**FIGURE 1:**
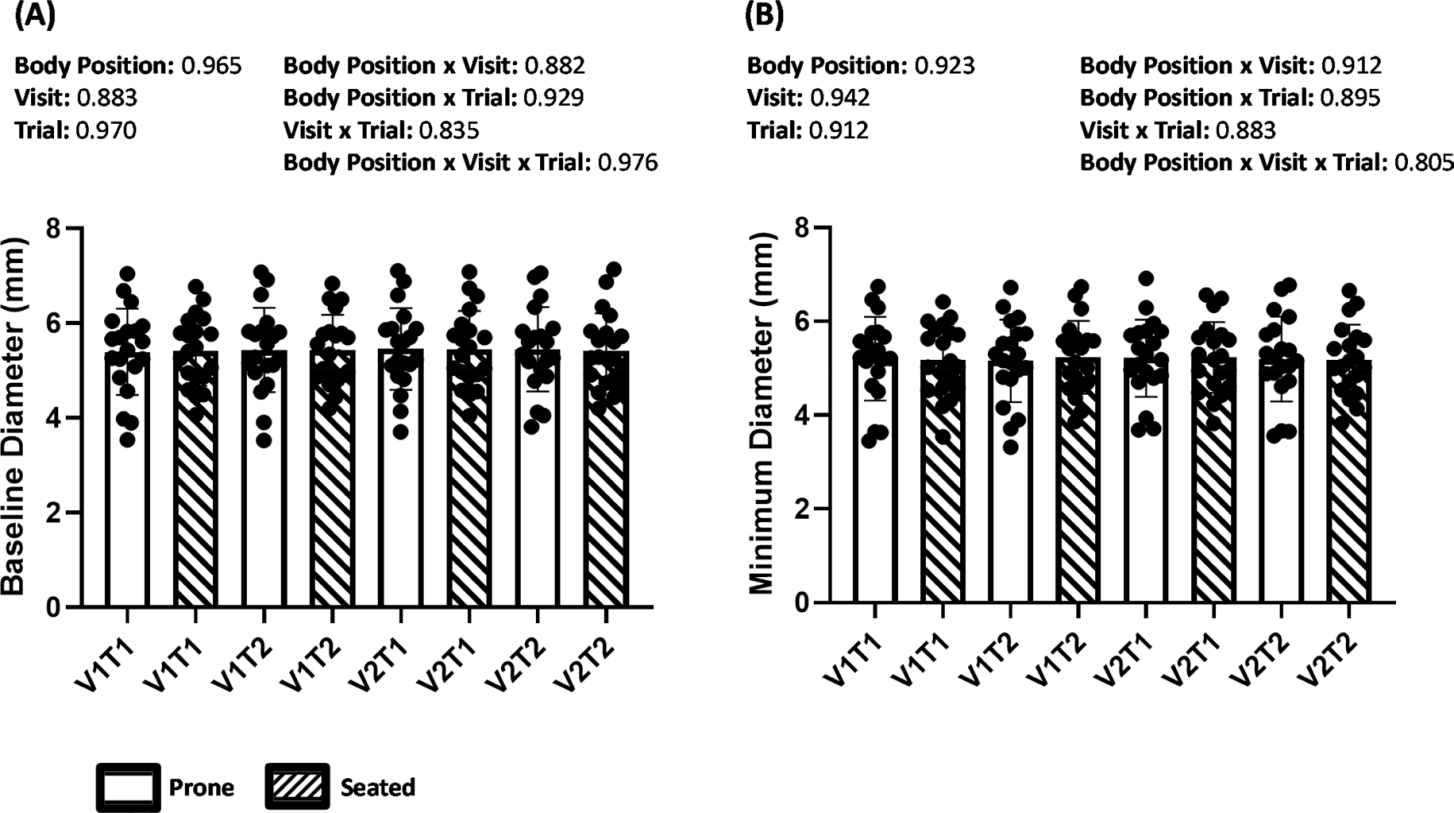
Baseline diameter (a) and minimum diameter (b) within each trial and across visits: 1st Visit 1st Trial (V1T1), 1st Visit 2nd Trial (V1T2), 2nd Visit 1st Trial (V2T1), and 2nd Visit 2nd Trial (V2T2). Three‐way analysis of variance *p* values for each main effect and interaction are listed above the bar graphs. Mean ± S.D.

Baseline diameter and minimum diameter during cuff inflation in the seated and prone positions within visits and between visits are represented in Figure 1. No significant main effects or interactions for body position, visit, or trial were found on baseline diameter or minimum diameter during cuff inflation using a 3‐way ANOVA (*p* > 0.05). There was no difference (Wilcoxon Signed Rank Test) between the seated (5.13 ± 2.98) and prone (4.95 ± 2.04) positions on %FMD (Z = ‐0.422, *p* = 0.673) (prone: median value 4.53 (IQR [3.69–6.21]) and seated: median value 4.70 (IQR [3.14–6.53])). Further, there was no difference (Wilcoxon Signed Rank Test) between the seated (5.70 ± 0.76) or prone (5.70 ± 0.91) positions on peak diameter (mm) (Z = ‐0.91, *p* = 0.927) (prone: median value 5.81 (IQR [5.19–6.12]) and seated: median value 5.70 (IQR [5.09–6.18])).

## 4 DISCUSSION

The main purpose of this study was to assess the reliability of the popliteal artery FMD measurement in the seated position within and between visits. Moreover, this study also focused on investigating the difference in %FMD between the prone and seated positions. A total of eight trials across two visits (four in the seated position and four in the prone position) were assessed. The findings indicate that the baseline diameter, peak diameter, minimum diameter, and %FMD of the popliteal artery had moderate‐to‐excellent reliability in the seated position both within and between visits. Although the baseline diameter, peak diameter, and minimum diameter had excellent reliability within and between visits in the prone position, %FMD demonstrated poor‐to‐moderate reliability both within and between visits in the prone position. Furthermore, there were no significant differences in baseline diameter or minimum diameter between body positions, visits, or trials. %FMD and the peak diameter post‐cuff deflation were not different between body positions.

This study reported ICC values to assess the reliability of popliteal artery FMD measures. The ICC values for baseline diameter, peak diameter following cuff deflation, and minimum diameter during cuff inflation all have excellent reliability (ICC > 0.9) in both positions (seated and prone position) within and between visits (Koo & Li, 2016). These results confirm findings from McLay (2012) in that popliteal artery FMD measurements made in the prone position demonstrate high reliability for baseline (ICC: 0.91) and peak (ICC: 0.86) diameters. This high reliability for the baseline and peak diameter has been supported in other studies examining other conduit arteries (Harris et al., 2007; West et al., 2004). For example, West et al. (2004) found that the baseline and peak diameter of the brachial artery had a small between visit coefficient of variation (2.7% and 2.5%, respectively). These findings are comparable to the reliability that was found for the popliteal artery in the present study. However, we expand upon the current literature knowledge base by demonstrating that baseline diameter, peak diameter, and minimum diameter demonstrate excellent reliability in the popliteal artery between and within visits in both the seated and prone positions.

The reliability for popliteal artery %FMD had moderate‐to‐good reliability in the seated position for both within and between visits. To our knowledge, this is the first study which reports on the reliability of the popliteal artery in the seated position. Given that numerous studies utilize this body position to assess FMD (Liu et al., 2021; O'Brien et al., 2020; Wu, 2022) and the clinical implications of the FMD technique, determining the reliability of FMD in the seated position is critical. The present study suggests that %FMD measured in the seated position has good reliability. On the other hand, %FMD measured in the prone position demonstrated less reliable ICC values of 0.60 and 0.25 within visit 1 and visit 2 trials, respectively. A range of ICC values from 0.35 to 0.74 was found in the prone position for between‐visit reliability, which indicates that reliability of the popliteal artery %FMD is poor‐to‐moderate (ICC < 0.75) in the prone position (Koo & Li, 2016). Interestingly, the present study found that baseline diameter and peak diameter had reliable ICC values, while %FMD did not. This was surprising given that %FMD is derived from the change in peak diameter to baseline diameter. West et al. (2004) and McLay (2012) report similar findings to the present study in the prone position. It is likely that the error in %FMD measurement is amplified due to the relatively small difference in the change in diameter from baseline to peak (%FMD). Thus, resulting in a worse ICC value for %FMD (McLay, 2012). The mechanism behind why the seated position but not the prone position had a good %FMD reliability is not known but should be investigated in future studies. However, this study appears to be the first to demonstrate that popliteal artery %FMD has good reliability in the seated position.

Vascular shear rate, which has an important role in stimulating vasodilation of blood vessels (Smieško et al., 1985), is reduced in the seated position compared to the supine position (Trinity et al., 2014). However, popliteal artery %FMD was not different between the seated and prone body positions. Soga et al. (2007) demonstrated similar findings in the brachial artery when comparing %FMD in the seated versus supine body position. To our knowledge, this study is the first to report in the popliteal artery that there is no statistically significant difference in %FMD between the seated and prone positions.

This study has some limitations, including that it was only conducted on people who were apparently healthy and self‐reported no relevant co‐morbidities. Although the participants fasted for 6 h prior to the visit, their diets before each visit were not standardized, which might have influenced the FMD measures. One study demonstrated that low salt consumption (50 mmol Na/d) resulted in a lower brachial artery FMD compared to the usual salt consumption (150 mmol Na/d) diet (Dickinson et al., 2009). Another study demonstrated increased brachial artery FMD in subjects who consumed a low‐carbohydrate diet for 12 weeks compared to a low‐fat diet (Volek et al., 2009). Thus, food that is consumed prior to the FMD test may impact %FMD.

To conclude, this study demonstrated that popliteal artery %FMD has moderate‐to‐good reliability for both trial‐retrial and visit‐to‐visit measurements in the seated position. However, poor‐to‐moderate reliability was demonstrated when %FMD was performed in the prone position. One implication of this study is that popliteal artery FMD can be performed in the seated position following recommended guidelines, which will help researchers conduct studies related to prolonged sitting or a sedentary lifestyle in the seated position. Moreover, given that popliteal artery %FMD was less reliable in the prone position may lead researchers and clinicians to reconsider using this body position to measure %FMD or in predicting cardiovascular disease risk in clinical trials.

## FUNDING INFORMATION

This project was not funded.

## CONFLICT OF INTEREST STATEMENT

The authors report no conflicts of interest.

## Data Availability

Data generated or analyzed during this study are available from the corresponding author upon reasonable request.
